# Thermometry*Read before the Maryland State Veterinary Medical Society.

**Published:** 1893-01

**Authors:** William Dougherty

**Affiliations:** Veterinarian


### THERMOMETRY.*

By William Dougherty, Veterinarian.

About twenty years ago I commenced the study of "Thermometry," and pursued it with considerable energy for four years in the training of race horses. I found it of considerable advantage to me as an aid to prognostication in the qualities of a colt for racing purposes.

My first observations were with colts about to be broken, or doing their first work, before engaging them in races. You must know that a colt is sometimes engaged in races to the value of two or three hundred thousand dollars, and it costs sometimes hundreds, and even thousands of dollars to enter a colt for so many stakes.

I commenced by taking the temperatures of colts before and after their work, that is, their ordinary work each day, and making a prognosis of the colt's future prospects in the racing world; also taking the temperature after they had been tried, as is customary to give yearling colts and fillies a trial of half a mile previous to the 1st of January of the year in which they start to race.

I pursued my observations up to four mile heats.

The last year I made some observations in the temperature of horses that had been prostrated from the heat. My attention was first called to the high elevation of temperature some years ago, but I never had an opportunity to positively find the true temperature until last summer. Last winter I had made for me a thermometer registering 120 degrees F. I could then register the correct temperature in horses that had been overheated during the excessive hot spell of last summer.

When I was studying veterinary medicine I was taught to believe that the temperature of 108 F. would destroy the blood corpuscles in a few hours.

You can imagine my feelings in treating a race mare (that had the year previous won about $30,000, and was herself valued at $25,000). She was in the stable that I was connected with.

One afternoon I was called to the other training stable; there were two on the farm, one for the old horses, the other for the colt

* Read before the Maryland State Veterinary Medical Society.

I had charge of the colts and studs. I found the mare had a chill. She was treated with aconite, mustard, warm clothing, bandages, &c. Following the chill she had a fever, and her temperature registered 108 degrees F. There was no veterinarian, that I knew of, nearer than 200 miles. I do not think I ever experienced in my life five hours with so much anxiety as those were, watching that mare.*

She made a complete recovery, and the following day you could not have told there had been anything the matter with her. It was this case that set me to thinking about the study of animal heat, or how high the temperature may run before death takes place, or at what point the blood corpuscle loses its formation. The next two cases were two horses in the same stable in the year 1880. One had a temperature of 107.2 degrees F., the other 108 degrees F., and in both cases lasted for seven days. They both recovered.

In 1889 I had several cases of sunstroke. My thermometer registered 110 degrees. I found that it would take one to two hours' treatment with cold applications before the temperature would come down to 110, or when the thermometer would register. I do not propose to say one word in this paper about the temperature of the horse in the ordinary diseases to which he is subject, as each disease has its own temperature, and as each and all of you are as well acquainted with it as I am. It is merely to the extraordinary cases that I wish to call your attention. [Exercise increases the temperature in all animals. It is not unusual for the temperature to raise to 108 degrees F. from work in hot weather. If a horse is led to one's yard for examination as to disease, do not depend upon the temperature until the animal has stood for at least an hour.]

Unfortunately for me, I have loaned my data of the early cases, and never had it returned. Therefore, what I will say will be from memory alone. First, I found that the colts that were the fattest, or in the best order, so to speak, were the ones that the temperature raised highest in. In their work, after they had become better conditioned, they could do more work with less elevation in the temperature than when they were gross. In yearlings, it would often occur that the temperature would rise to 104 from very little work. In three (3) year olds, you could work a horse a full

* Dr. Dougherty's paper recalls to me a two-year-old colt which, after a trial, had a temperature of 112 degrees F. for three hours, with a congestion of the lungs, which dropped after a bleeding of three quarts and a one drachm dose of Kermes mineral.—HUIDEKOPER.

mile at speed and his temperature would only reach 104. In other horses, you could work 1½ miles with the same temperature, and with the same effects, in the same horse in different conditions. The mare "Bessie Lee" ran a four mile heat race at Baltimore, in the month of October 1874. In the first heat the temperature registered only 104. In the second heat, 106. In the third heat, her rectum was so open that I could not take her temperature [This fact teaches a clear lesson as to the effects of exhaustion which are often considered as symptoms of disease.] My observations were that colts whose temperature did not run high during their first work, were the ones you could expect the most of, in after life as a race horse. That is, everything is taken in consideration that the colts were in good health at the time when the temperature was taken. For instance, take two horses that were in the pink of condition to the trainer's eye. Give them a trial together—for one mile, say in 1.50. One horse registers 103, the other 102½. The one that registers the lowest is the one that would go the farthest, with the least exertion, and the one that would stand the hardest drive at the finish of a race. Low temperatures often occur in diseases. The lowest I ever saw in a horse was 96 F. That was in a case of ruptured stomach, which died in a few hours after I saw it.

The following memorandum was taken last summer of three cases that I came in contact with. One was a case of my own that recovered. The other two, one of which had no treatment. It died in five minutes from the time it fell. The third was that of a hack horse on Charles Street. It died.

First. Gray gelding worked in a street car, temperature 114 degrees F. Died in five minutes.

Second. Bay horse. Temperature when I found him was 112; in 15 minutes 112; in 30 minutes 111; in 45 minutes 109½; in 60 minutes 107; in 75 minutes 105; in 90 minutes 103½.

Then taken to the stable and made good recovery.

He was treated with abundance of cold water for seventy-five minutes, until his temperature reached 105 degrees, then he had stimulants administered.

No. 3 was the hack horse. I got off a car and took his temperature, which was 110½ degrees. He looked as if he ought to recover. They were feeding him aconite. I afterwards learned that he died.

